# Maternal mindful eating as a target for improving metabolic outcomes in pregnant women with obesity

**DOI:** 10.52586/5048

**Published:** 2021-12-30

**Authors:** Karen L Lindsay, Jasper Most, Kerrie Buehler, Maryam Kebbe, Abby D Altazan, Leanne M Redman

**Affiliations:** 1 Susan Samueli Integrative Health Institute, University of California Irvine, Costa Mesa, CA 92696, USA,; 2 Department of Pediatrics, Susan and Henry Samueli College of Health Sciences, University of California Irvine, Irvine, CA 92697, USA,; 3 Zuyderland Medical Center, Sittard/Geleen, 6162 BG, The Netherlands,; 4 Reproductive Endocrinology and Women’s Health Laboratory, Pennington Biomedical Research Center, Baton Rouge, LA 70808, USA,; 5 Department of Medicine, Susan and Henry Samueli College of Health Sciences, University of California Irvine, Irvine, CA 92697, USA

**Keywords:** Pregnancy, Maternal obesity, Mindful eating, Dietary inflammatory index, Gestational weight gain, Adiposity, Insulin resistance, Ghrelin

## Abstract

**Background:**

Maternal diet and eating behaviors have the potential to influence the metabolic milieu in pregnancies complicated by obesity, with implications for the developmental programming of offspring obesity. Emerging evidence suggests that mindfulness during eating may influence metabolic health in non-pregnant populations, but its effects in the context of pregnancy is less well understood. This study explored the individual and combined effects of mindful eating and diet quality on metabolic outcomes among pregnant women with obesity.

**Methods:**

In 46 pregnant women (body mas index >30 kg/m^2^) enrolled in the MomEE observational study, mindful eating (Mindful Eating Questionnaire, MEQ) and energy-adjusted dietary inflammatory index (DII, from 7 days of food photography) was assessed at two time points and the mean pregnancy values computed. Rate of gestational weight gain (GWG) and fat mass gain per week were determined from measured weight and body composition using a three-compartment method, respectively, at each assessment. Homeostasis Model Assessment of Insulin Resistance (HOMA-IR) and ghrelin concentrations were determined from fasting blood samples in late gestation (35–37 weeks). Linear regression was used to examine the association of the MEQ and its subscales (where higher values indicate more mindful eating) with metabolic outcomes, adjusting for covariates: maternal age, pregravid body mass index, race, parity, DII. The effects of the MEQ*DII interaction was also tested.

**Results:**

Total MEQ scores were not associated with rate of weight or fat mass gain, although greater distracted eating behavior was associated with greater adiposity gain (weight and fat mass). Mindful eating was inversely associated with insulin resistance, although this was attenuated to non-significance after additional adjustment for GWG. Total MEQ and the external eating subscale was significantly inversely associated with fasted ghrelin, such that less tendency to eat under the influence of external cues was associated with lower ghrelin concentrations. After false discovery rate adjustment for multiple testing, only the association of the total MEQ and external eating subscale with ghrelin levels trended towards significance. The DII was not associated with MEQ scores or outcome variables, nor did it moderate the effect of MEQ on any of the outcomes.

**Conclusion:**

This study generates early evidence to suggest that mindful eating holds potential as a tool to improve metabolic health outcomes in pregnant women with obesity, although further research is required on this topic. Prenatal lifestyle interventions should consider including mindfulness during eating to determine its efficacy for reducing adverse pregnancy and offspring health outcomes associated with maternal obesity.

## 1. Introduction

The prevalence of maternal obesity in pregnancy is steadily increasing in the US [1] and worldwide [2], which has significant implications for the intergenerational transfer of obesity risk. Pre-pregnancy obesity is associated with heightened insulin resistance, hyperglycemia, hypertriglyceridemia, and development of gestational diabetes mellitus (GDM), creating an adverse metabolic milieu for the developing fetus [3]. The excess availability of maternal metabolic substrates promotes fetal overgrowth, higher infant birthweight and adiposity, and risk for childhood obesity [4]. The combination of maternal obesity and excess gestational weight gain (GWG) has been shown to further exacerbate risk for offspring obesity [5]. However, gain in maternal adiposity (fat mass) rather than absolute weight gain, is a more sensitive predictor of the metabolic milieu that influences fetal growth and infant adiposity [6–8], yet this measure is rarely considered in clinical research.

Clinical trials targeting improvements to the maternal diet among pregnant women with overweight and obesity have largely focused on the composition of the diet rather than eating behaviors, including mindful attention, awareness, and limiting distractions during meals. Eating mindlessly under the influence of external cues and the rewarding value of hyperpalatable foods overrides homeostatic signals for hunger and satiety, leading to overconsumption and weight gain [9]. While classical dietary modification approaches have demonstrated some success in limiting GWG [10], they have largely failed to prevent GDM, large-for-gestational age births, [11, 12] or to reduce childhood adiposity [13]. Past trials have also neglected to measure effects on maternal fat mass gain. This suggests that modest changes in maternal energy intake are insufficient to prevent adverse pregnancy outcomes, and that diet quality and its behavioral influences must be considered more prominently.

Currently, there is lack of consensus on what constitutes the optimal diet for ensuring a healthy metabolic state in pregnancy [14]. The dietary inflammatory index (DII), a measure of the inflammatory potential of the diet [15], may hold promise as a new tool for informing future prenatal dietary interventions. Recent observational studies in pregnancy cohorts spanning the full range of maternal BMI demonstrate a positive association between the DII and levels of inflammatory cytokines [16], incidence of GDM [17], and neonatal adiposity [18]. While maternal obesity is associated with consuming a diet of higher inflammatory potential [16], the metabolic effects (e.g., glucose intolerance, gain in fat mass) of this dietary pattern among pregnant women exclusively with obesity has not yet been studied.

The maternal diet and metabolic milieu in pregnancy are also influenced by psychological factors [19, 20], thereby representing an important and complementary pathway to include in interventions designed to improve maternal and offspring health. Yet, a paucity of prenatal intervention studies targeting maternal psychological state report on metabolic or diet-related outcomes [21]. Mindfulness and mindful eating have been found to exert beneficial effects on eating behaviors [22] and glycemic control [23] in non-pregnant, high metabolic risk populations. The potential for mindfulness approaches to influence weight and fat mass gain and other metabolic outcomes in the context of pregnancy requires further investigation.

The aim of this study is to determine the individual and combined associations of mindful eating and the DII across pregnancy on total weight and adiposity gain, as well as markers of insulin resistance [homeostasis model assessment of insulin resistance (HOMA-IR)] and hunger levels (ghrelin) in late gestation, among pregnant women with obesity.

## 2. Materials and methods

### 2.1 Study design

This is a secondary analysis of a prospective observational study designed to assess determinants of GWG in pregnant women with obesity [24, 25]. Maternal assessments were performed between 13 and 16 weeks (‘early’), and between 35 and 37 weeks (‘late’) gestation. The study was approved by the Institutional Review Board of Pennington Biomedical Research Center and University of California, Irvine. Participants provided written informed consent prior to participating.

### 2.2 Participants and recruitment

Women aged 18 to 40 years, with obesity (BMI ≥30 kg/m^2^) at screening (<15 weeks of gestation), and a confirmed singleton, viable pregnancy were eligible to enroll in the study. Women were excluded for recent history of smoking, alcohol or drug use, pre-existing hypertension (i.e., systolic blood pressure >160 mmHg and diastolic blood pressure >110 mmHg), diabetes (HbA1c ≥6.5%), HIV or AIDS, severe anemia (hemoglobin <8 g/dL and/or hematocrit <24%), contraindications to MRI (implanted metal objects, claustrophobia), prior or planned (within 1 year of expected delivery) bariatric surgery, and psychological or eating disorders. Study participants were recruited from January 2015 to January 2017 through community and social media advertisements and referrals by local obstetricians. Demographic information such as age, race/ethnicity, and parity were collected by maternal self-report at enrollment and confirmed by medical chart review.

### 2.3 Maternal anthropometrics and adiposity

Early pregnancy BMI was calculated from maternal weight and height measured at screening (<15 weeks’ gestation). Maternal weight and body composition were measured after an overnight fast during both early and late pregnancy assessments. Total GWG was computed as the difference between measured weight at late gestation and enrollment weight, and rate of GWG per week was determined by dividing total GWG by the number of gestational weeks between measurements. Body fat mass was calculated at each visit using body weight, body volume by plethysmography (BODPOD®, COSMED, Concord, CA), and body water (mean estimate of using zero-intercepts of ^2^H and ^18^O-isotopes, mean ND/NO = 1.0xx) [26]. The rate of change in fat mass per week was computed using the same approach as rate of GWG.

### 2.4 Metabolic biomarkers

Fasting blood samples were collected in the morning following a standardized dinner and 12-hour fast in late gestation. Blood was drawn into a red-top tube without additive, transported on ice and centrifuged at 3500 rpm for 15 minutes for measurement of glucose (DXC600; Beckman Coulter Inc., Brea, CA, USA) and insulin (ELISA, Immulite 2000; Siemens, Broussard, LA, USA). A second sample was drawn into a purple-top EDTA tube, mixed with protease cocktail inhibitor, transported on ice and centrifuged at 3000 rpm for 15 minutes for measurement of total and active ghrelin (RIA; MilliporeSigma, Burlington, MA, USA). HOMA-IR was computed according to the formula: [fasting glucose (mg/dL)*fasting insulin (uU/mL)]/405 [27].

### 2.5 Mindful eating questionnaire

Mindfulness towards eating was assessed with the Mindful Eating Questionnaire (MEQ) which has been validated in pregnant women [28]. The MEQ explores mindfulness across five subscales, including disinhibition, awareness, external cues, emotional response, and distraction [29]. Mindful eating refers to an unbiased awareness of sensations around eating. Disinhibition measures the inability to stop eating even when full. The awareness subscale measures an individual’s awareness of the sensory aspects of eating. Distraction refers to the tendency to think about other things and rush while eating. The external cues subscale refers to eating in response to environmental cues, and emotional response refers to eating in response to negative emotions. Higher scores within each subscale and in the total score are indicative of mindful eating.

### 2.6 Dietary inflammatory index

Participants captured their dietary intake in real-time using remote food photography over seven days at each assessment, as previously described [30, 31]. Reported energy intake was compared to total daily energy expenditure measured by doubly labeled water and days with reported energy that was <60% of energy expenditure were excluded. Dietary intake data were analyzed for macronutrient and micronutrient content and average values across the reporting days at each assessment were computed. The adapted DII was then computed according to methods previously described [32]. Briefly, the residual method was applied to retrieve energy-adjusted daily intakes of saturated fatty acids, trans fatty acids, monounsaturated fatty acids, omega-3 fats, omega-6 fats, cholesterol, carbohydrates, fiber, protein, vitamin A, beta-carotene, vitamin C, vitamin D, vitamin E, thiamine, riboflavin, niacin, folate, vitamin B6, vitamin B12, iron, magnesium, selenium, zinc, caffeine, and ethanol. Intakes of other components that were included in the original DII (eugenol, flavan-3-ol, flavones, flavanones, isoflavones, anthocyanidins, quercetin, tea, garlic, ginger, saffron, pepper, thyme or oregano, rosemary, onions, turmeric) were unavailable from our dietary dataset and, therefore, were not taken into account to investigate the inflammatory potential of the diet in this study. Energy-adjusted intakes for each parameter were subsequently standardized by subtracting the cohort mean intake from the intake of each individual and dividing by the cohort standard deviation. The resulting *z*-scores for each parameter were multiplied by their respective inflammatory weights, as defined by Shivappa *et al*. [15], to generate an adapted DII value for each food component. Finally, the individual parameter scores were summed to create one final DII score, where negative values indicate an anti-inflammatory potential of the diet and positive values indicate a pro-inflammatory dietary potential.

### 2.7 Statistical analysis

Descriptive statistics were used to describe maternal characteristics and the distributions of the predictor (MEQ, DII) and outcome variables (GWG/week, fat mass gain/week, HOMA-IR, ghrelin). The distribution for all variables was inspected using histograms and HOMA-IR and ghrelin were log-transformed for normality. As there was no significant difference in MEQ and DII scores between early and late gestation, mean values were computed and used in analyses. Bivariate associations between the mean pregnancy MEQ score and its subscales, DII, and baseline maternal characteristics were analyzed by Pearson’s correlations. Associations between the total and subscale MEQ scores and outcome variables were tested by linear regression, before and after adjusting for *a priori*-defined covariates (maternal age, early-pregnancy BMI, parity, race, DII). To correct for multiple testing, a false discovery rate (FDR) of less than 5% was used as the significance level [33]. A FDR correction was applied to the analyses of the MEQ total score with outcome variables (4 tests) and to the analyses of the 5 MEQ subscales with 4 outcome variables (20 tests). To test effect modification of mindful eating on outcome variables by the DII, the product of the DII and MEQ scores at each assessment was computed and entered into the linear regression models. All analyses were performed with SPSS for Macintosh, version 26.0 (SPSS Inc., Chicago, IL, USA) and results were considered statistically significant at the level of *p* < 0.05.

## 3. Results

### 3.1 Maternal characteristics and anthropometry

Seventy-two participants were enrolled in the parent study. For this analysis, complete data on MEQ, dietary intakes, and metabolic outcomes were available for forty-six participants (Table 1). The majority of our cohort was either non-Hispanic Black (47%) or non-Hispanic White (45%), which is representative of the maternal population in the state of Louisiana [34]. The mean BMI on enrollment was 36.5 ± 5.2 kg/m^2^ and the mean GWG was 9.9 ± 6.0 kg (range: −4.2 to 24.9 kg). The mean change in fat mass across gestation was a gain of 0.7 ± 3.4 kg, with a range from −6.4 to +7.2 kg.

Across gestation, 28% of participants reported a decrease in their total MEQ score (mean difference: −0.18 ± 0.06), 35% increased their score (0.23 ± 0.11) and 37% were stable (0.01 ± 0.05). For the DII score, 30% decreased (i.e., consumed a more anti-inflammatory diet; mean difference −2.34 ± 1.09), 27% increased (i.e., consumed a more pro-inflammatory diet; 2.37 ± 0.99), and 43% remained stable from early to late gestation (−0.07 ± 0.51). On average, there was no significant change in either MEQ or DII scores across pregnancy. The MEQ score ranged from 2.1 to 3.6 and the range of the DII was −4.6 to +5.7. Maternal characteristics (age, pregravid BMI, parity) were not correlated with either the DII or MEQ, but participants of Black race consumed a more pro-inflammatory prenatal diet compared to non-Black participants (1.0 ± 1.3 vs. −0.6 ± 2.7, *p* = 0.02). Fat mass at baseline was positively correlated with maternal age (r = 0.4, *p* = 0.002) and pregravid BMI (r = 0.9, *p* < 0.001), and was non-significantly lower among women of Black versus non-Black race (43.2 vs 46.1 kg, *p* = 0.33).

### 3.2 Association of mindful eating with adiposity gain

**Fig. 1.**
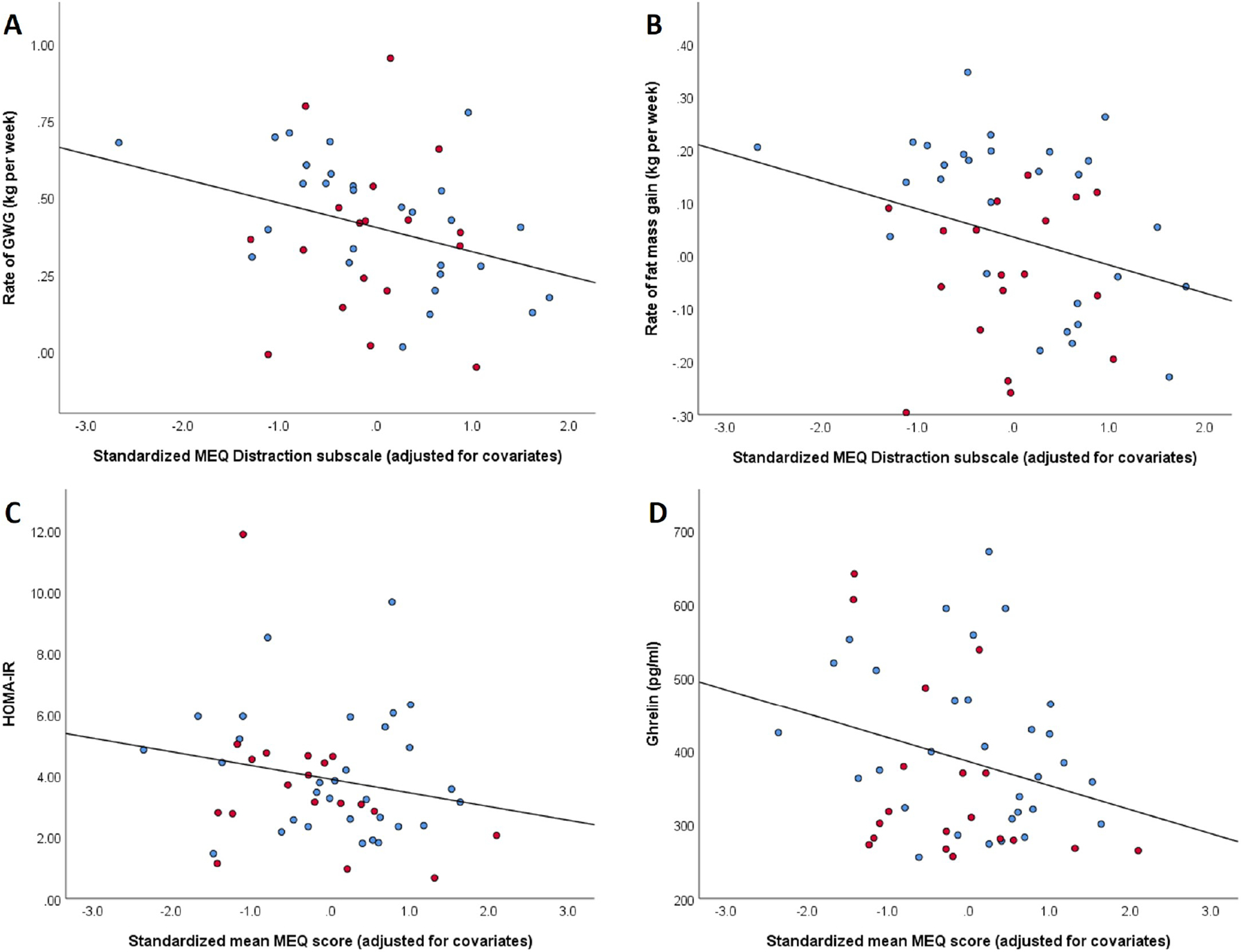
Association of mindful eating with metabolic outcomes in pregnant women with obesity, identified by race category. Linear association of the mindful eating distraction subscale with (A) rate of gestational weight gain and (B) rate of fat mass gain, and linear association of the total mindful eating score with (C) insulin resistance and (D) fasting ghrelin concentration. Independent variables (x-axes) are adjusted for maternal age, early pregnancy BMI, parity, and race. Red markers represent participants of Black or African-American race and blue markers represent participants of non-Black or African-American race.

The mindful eating total score was not associated with rate of weight or fat mass gain, adjusting for covariates (Table 2, Ref. [35]). The distracted eating subscale was significant, such that more distracted eating behavior was associated with greater total weight gain (B = −0.13, uncorrected *p* = 0.03) and the gain in fat mass (B = −2.07, uncorrected *p* = 0.02) (Table 3, Ref. [33]; Fig. 1). However, this association was not significant after FDR correction for multiple testing.

### 3.3 Association of mindful eating with insulin resistance

The total MEQ score was inversely associated with HOMA-IR, such that more mindful eating was associated with less insulin resistance (Table 2, Fig. 1). This association with the total MEQ score did not hold significance after FDR correction, and none of the MEQ subscales were associated with insulin resistance (Table 3). Given the observed associations between the MEQ and adiposity gain, we conducted a sensitivity analysis to additionally control for rate of GWG, as this variable may be on the causal pathway of developing insulin resistance in late pregnancy. This attenuated the association of the total MEQ score (B = −0.21, *p* = 0.13) and emotional eating score (B = −0.06, *p* = 0.08) with HOMA-IR.

### 3.4 Association of mindful eating with diet quality and hunger signals

**Fig. 2.**
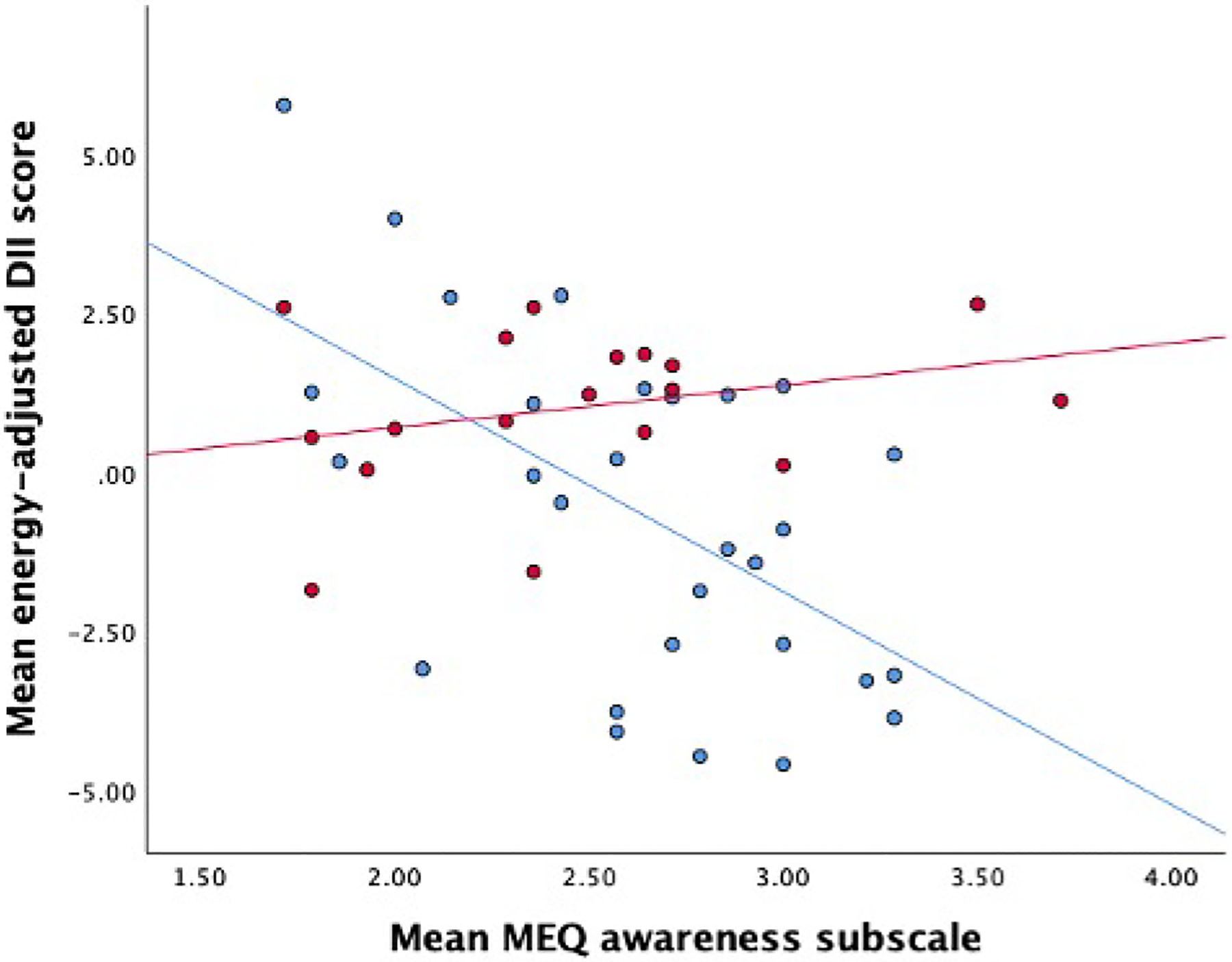
Association of mindful eating with the dietary inflammatory index in pregnant women with obesity, identified by race category. Linear association of the mindful eating awareness subscale with the dietary inflammatory index, stratified by race category. Red markers represent participants of Black or African-American race and blue markers represent participants of non-Black or African-American race. Fit lines represent the direction of association for each race category.

The MEQ total score was not correlated with the DII (r = −0.1, *p* = 0.46) but lower awareness of the sensory aspects of eating subscale was associated with a more pro-inflammatory diet (r = −0.3, *p* = 0.02). When stratified by maternal race, the correlation between the awareness subscale and the DII was only evident among women of non-Black race (r = −0.57, *p* = 0.001; Fig. 2). Total MEQ scores were inversely associated with fasting ghrelin levels (Table 2, Fig. 1), driven by the external eating subscale (Table 3), such that less tendency to eat under the influence of external cues was associated with lower ghrelin (B = −0.13, uncorrected *p* = 0.003). These associations between between total MEQ and external eating subscale scores with ghrelin levels held a trend towards significance after FDR correction. In a sensitivity analysis, additional adjustment for rate of GWG attenuated the association with the total MEQ score (B = −0.11, *p* = 0.09), but not for the external eating subscale (B = −0.14, *p* = 0.002).

The DII was not associated with any of the outcome variables in regression models (Table 2), nor did it moderate the effect of MEQ on any of the outcome measures (*p* > 0.05 for interaction term in all models).

## 4. Discussion

Mindful eating is a branch of mindfulness practice that is accessible to all individuals and aims to bring awareness and attention to the process of eating, while minimizing any distractions or thought processes unrelated to eating. In this way, mindful eating promotes healthy eating behaviors that may be conducive to weight management and improved metabolic health [36, 37]. Although the results of this study become non-significant after correction for multiple testing, the unadjusted results provide initial insight to the potential for aspects of mindful eating to beneficially influence the metabolic milieu in pregnant women with obesity. This exploratory analysis adds to a sparse literature on the relationship between mindful eating and metabolic health outcomes. Thus, the discussion focuses on the results from the unadjusted analysis in order to provide a basis for future research to test investigate whether mindful eating during pregnancy may exert positive metabolic health effects.

Mindfulness during eating was modestly associated with less insulin resistance and lower ghrelin levels measured cross-sectionally in late gestation, while less distraction during eating was associated with a lower rate of gain in weight and fat mass. The effects of mindful eating on insulin resistance appeared to be driven by the attenuated adiposity gain, while the effects on ghrelin concentrations were somewhat independent of this effect. Interestingly, the DII was not associated with any outcome measure, nor did it moderate the associations between mindful eating and the metabolic outcomes.

The underlying mechanisms that link mindful eating behaviors to improved metabolic health outcomes are potentially related to appetite regulation and biological stress pathways. Ghrelin is an appetite-stimulating hormone produced in the stomach and higher circulating concentrations are associated with hedonic eating and preference for sweet foods [38, 39]. More mindfulness during eating was associated with lower hunger signals measured from fasting samples, and this appeared to be driven by the external eating subscale. These findings suggest that a lower susceptibility to external food cues, such as food marketing, may contribute to decreased appetite in pregnant women with obesity. The potential to elicit appetite-regulation via decreased plasma ghrelin in response to a prenatal mindful eating intervention remains to be determined.

It is also possible that mindfulness around eating either promotes, or is a response to, improved psychological states (e.g., low stress, positive affect), which is generally associated with lower circulating levels of the stress hormone cortisol. As a catabolic hormone, cortisol promotes elevated blood glucose to fuel the stress response through increased glycogenolysis, gluconeogenesis, and reduced postprandial glucose clearance [40–42]. Thus, psychological distress may contribute to insulin resistance over time, regardless of diet quality. This has been demonstrated in a prenatal study that investigated the interactive effects of negative mood state and diet quality on levels of HOMA-IR in the third trimester, and found that heightened negative mood overshadowed any beneficial effects of a healthy Mediterranean dietary pattern on insulin resistance [20]. Lower maternal stress levels may also contribute to lower plasma ghrelin among those with higher mindful eating scores, as this hormone has been found to increase under repeated or chronic stress [43], possibly in direct response to elevated cortisol levels [44]. In this way, ghrelin may increase motivation to eat under stress, a pattern that is commonly observed in individuals with overweight and obesity [45], and may contribute to poor metabolic outcomes in pregnant women [21]. Although maternal stress was not assessed in our study, we propose this to be a probable factor mediating the association between mindful eating, weight and adiposity gain, hunger levels, and insulin resistance.

In our cohort of 46 pregnant women with obesity ranging from 30.2 to 57.1 kg/m^2^, the DII score range was similar to that reported in a previous prenatal population using the energy-adjusted DII approach (range: −5.0 to +5.0) [46]. Total mindful eating scores and the DII were not correlated with one another. However, participants who consumed a more anti-inflammatory diet reported greater awareness of the sensory aspects of eating (i.e., attention to taste, smell, texture), which was particularly evident among those of non-Black race. Previously, the MEQ awareness subscale was shown to significantly contribute to fruit and vegetable consumption among pregnant women [47]. Together, this suggests that this aspect of mindful eating practice may hold potential to positively influence the maternal diet, although the effects may be influenced by ethnic and cultural factors. A recent systematic review found insufficient evidence for any beneficial effects of mindful eating on energy intake or diet quality [48], although we note that there was a high risk of bias across included studies and no prenatal studies were included. Pregnancy is a unique life stage in which mothers may be more receptive to health behavior change for the benefit of their future offspring, and bringing greater awareness to quantity and quality of food consumed through mindful eating approaches could potentially support improved nutritional intake. Thus, prenatal intervention studies are required to systematically test the potential benefits of mindful eating practices on maternal dietary quality in diverse cohorts, and whether any effects may translate to improved biological markers or perinatal outcomes.

There is limited literature to date reporting associations of mindfulness in pregnancy on GWG and metabolic markers. Epel *et al*. [49] conducted a randomized controlled trial of a prenatal mindfulness intervention, which incorporated mindful eating, among low-income women with overweight and obesity. They reported no beneficial effect on absolute GWG, although a higher proportion of women in the intervention group gained weight below the recommendations for their pre-pregnancy BMI category. Furthermore, in a subset of women (n = 141/209) with available data from a 1-hour glucose challenge test, the intervention was associated with a lower incidence of impaired glucose tolerance compared to control [49]. Our findings with respect to lower rate of GWG and less insulin resistance with less distracted eating behavior, contributes to this sparse literature regarding the potential beneficial effects of prenatal mindfulness training for metabolic health outcomes among women at heightened risk of glucose intolerance.

Our results are also supported by evidence from the non-pregnancy literature. In the context of diabetes, a randomized clinical trial that compared the effects of a 3-month mindful eating intervention to a traditional self-management program (nutrition counseling, exercise, self-monitoring blood glucose) found that both interventions exerted comparable and significant improvements to glycemic control measured by hemoglobin A1c [23]. This indicates that mindful eating may be an equally effective approach to traditional methods for diabetes management. Conclusions from a recent meta-analysis and integrative review support the efficacy of mindful eating interventions for weight loss among non-pregnant individuals with overweight and obesity, demonstrating them to be at least comparable to [50], if not modestly more effective than [51], conventional diet and exercise approaches. However, to our knowledge, no prenatal intervention study has specifically examined the effects of mindful eating alone on GWG or associated metabolic outcomes. This is an important question to investigate as it is possible that pregnant women are more receptive to the compassionate approach promoted by mindful eating techniques, as opposed to prescribed dietary and exercise regimens that do not necessarily address the underlying psychological states that drive eating behavior.

Although the MEQ total score was not associated with weight and adiposity gain across gestation in our study, the distracted eating subscale emerged as a factor related to these outcomes. Items in the MEQ that contribute to the distracted eating subscale include “I eat so quickly that I don’t notice what I am eating” and “I think about things I need to do while I am eating”. In a meta-analysis of studies in non-pregnant individuals that manipulated awareness, memory and attentiveness while eating, distracted eating was found to be the strongest determinant for quantity of food consumed in both the immediate term and later in the day [52]. Thus, future prenatal interventions that focus on mindful eating and emphasize attentiveness while eating are warranted to determine the effects on adiposity gain and biological markers of metabolic health.

Strengths of this study include the focus on maternal obesity from a diverse prenatal cohort; detailed characterization of maternal metabolic milieu using biomarkers and direct measurement of adiposity, as well as GWG; and characterization of dietary composition and eating behaviors using indices that have not yet been studied in a prenatal cohort exclusively with obesity. Study limitations include the small sample size which limited our ability to further interrogate differential effects by race/ethnicity, and may be underpowered to detect significant effects of MEQ on metabolic outcomes. The observational study design also precludes the ability to determine causation for the observed associations. As women with psychological or eating disorders were excluded from the study, the results may not be generalizable to such patient populations.

## 5. Conclusions

In conclusion, this study suggests mindful eating as a potential tool to improve metabolic health outcomes in pregnant women with obesity, although larger studies with adequate power are required to determine if these associations can be replicated. The potential mechanisms underlying these outcomes include improved recognition of hunger and satiety signals that may moderate total energy intake, and reduced biological stress signals that can influence glucose metabolism and energy storage as well as food cravings. Future prenatal lifestyle interventions should incorporate or focus on mindfulness during eating to determine the efficacy of this approach for reducing the risk of adverse maternal and offspring health outcomes associated with pregravid maternal obesity.

## Figures and Tables

**Table 1.** Descriptives of maternal baseline characteristics, anthropometry, biomarkers, diet and mindful eating scores.

Maternal Characteristic	Mean ± SD
Maternal age (years)	28.3 ± 4.9
Pre-pregnancy BMI (kg/m^2^)	36.5 ± 5.2
Class 1 obesity [N (%)]	26 (43.3)
Class 2 obesity [N (%)]	22 (36.7)
Class 3 obesity [N (%)]	12 (20.0)
Primiparous [N (%)]	29 (48.3)
Race [N (%)]	
White	27 (45.0)
Black	28 (46.7)
Asian	1 (1.7)
Other	4 (6.7)
Mean pregnancy MEQ total score	2.9 ± 0.3
MEQ Awareness	2.6 ± 0.5
MEQ Distraction	3.1 ± 0.6
MEQ Disinhibition	3.2 ± 0.5
MEQ Emotional	3.3 ± 0.5
MEQ External	2.3 ± 0.5
Mean pregnancy DII	−0.01 ± 2.4
Total GWG (Kg)	9.9 ± 6.0
Rate of GWG (kg/week)	0.4 ± 0.2
Rate of fat mass gain (kg/week)	0.04 ± 0.2
Biomarkers in late gestation	Median (IQR)
Fasting glucose (mg/dL)	85.5 (13.25)
Fasting insulin (mU/mL)	17.1 (9.5)
HOMA-IR	3.5 (2.3)
Ghrelin (pg/mL)	364.0 (184.0)

BMI, body mass index; DII, dietary inflammatory index; GWG, gestational weight gain; HOMA-IR, homeostasis model assessment of insulin resistance; IQR, interquartile range; MEQ, mindful eating questionnaire.

**Table 2.** Main effects of MEQ and DII on gestational metabolic outcomes.

	Beta	Std. Error	95% CI		*p*-value	Adj R2	*p*-adj*
**Rate of GWG**							
MEQ total (unadjusted)	−0.164	−0.119	−0.403	0.076	0.175		
DII (unadjusted)	−0.010	0.015	−0.039	0.020	0.513		
Adjusted model:							
MEQ total	−0.129	0.108	−0.348	0.089	0.239	0.243	0.319
DII	0.006	0.014	−0.023	0.034	0.683		
Parity	−0.136	0.038	−0.213	−0.060	0.001		
Pre-pregnancy BMI	−0.020	0.007	−0.033	−0.006	0.005		
Age	0.010	0.007	−0.004	0.024	0.177		
Race	0.051	0.071	−0.092	0.194	0.476		
**Rate of fat mass gain**							
MEQ total (unadjusted)	−0.099	0.084	−0.268	0.069	0.242		
DII (unadjusted)	−0.007	0.010	−0.028	0.014	0.469		
Adjusted model:							
MEQ total	−1.193	1.519	−4.265	1.879	0.437	0.329	0.437
DII	0.174	0.199	−0.229	0.577	0.388		
Parity	−0.043	0.044	−0.131	0.045	0.331		
Pre-pregnancy BMI	0.011	0.008	−0.004	0.026	0.147		
Age	0.007	0.008	−0.009	0.023	0.363		
Race	−0.041	0.083	−0.208	0.127	0.625		
**HOMA-IR**							
MEQ total (unadjusted)	−0.152	0.115	−0.382	0.079	0.193		
DII (unadjusted)	0.002	0.015	−0.029	0.032	0.922		
Adjusted model:							
MEQ total	−0.281	0.127	−0.539	0.024	0.033	0.097	0.132
DII	0.013	0.016	−0.020	0.046	0.425		
Parity	−0.037	0.045	−0.129	0.055	0.418		
Pre-pregnancy BMI	0.012	0.008	−0.004	0.027	0.150		
Age	0.009	0.008	−0.008	0.025	0.282		
Race	−0.014	0.085	−0.186	0.158	0.871		
**Ghrelin**							
MEQ total (unadjusted)	−0.141	0.056	−0.253	−0.029	0.015		
DII (unadjusted)	−0.010	0.008	−0.026	0.005	0.182		
Adjusted model:							
MEQ total	−0.131	0.063	−0.257	0.004	0.043	0.131	0.086
DII	−0.011	0.008	−0.028	0.005	0.161		
Parity	0.007	0.022	−0.037	0.051	0.756		
Pre-pregnancy BMI	−0.001	0.004	−0.008	0.007	0.848		
Age	−0.006	0.004	−0.014	0.002	0.123		
Race	−0.055	0.041	−0.138	0.028	0.188		

BMI, body mass index; DII, dietary inflammatory index; DV, dependent variable; GWG, gestational weight gain; HOMA-IR, homeostasis model assessment of insulin resistance; MEQ, mindful eating questionnaire.

* Adjusted *p*-value using the false discovery rate method [35].

**Table 3.** Association of MEQ subscales with gestational metabolic outcomes.

	Beta	Std. Error	95% CI		*p*-value	Adj R2	*p*-adj*
Rate of GWG							
Awareness	−0.006	0.066	−0.140	0.129	0.934	0.216	1.000
Distraction	−0.134	0.060	−0.254	−0.013	0.030	0.305	0.200
Disinhibition	−0.065	0.071	−0.208	0.078	0.365	0.232	0.730
Emotional	−0.059	0.061	−0.183	0.065	0.343	0.234	0.762
External	0.060	0.075	−0.092	0.212	0.429	0.228	0.660
Rate of fat mass gain							
Awareness	−0.172	0.924	−2.040	1.696	0.853	0.319	1.000
Distraction	−2.068	0.816	−3.719	−0.417	0.015	0.415	0.150
Disinhibition	0.082	0.993	−1.926	2.091	0.934	0.319	1.000
Emotional	0.033	0.865	−1.716	1.782	0.970	0.319	1.000
External	0.228	1.054	−1.904	2.360	0.830	0.319	1.000
HOMA-IR							
Awareness	−0.066	0.079	−0.225	0.093	0.406	0.002	0.738
Distraction	−0.071	0.087	−0.246	0.104	0.416	0.001	0.693
Disinhibition	−0.143	0.083	−0.311	0.025	0.092	0.056	0.307
Emotional	−0.141	0.074	−0.291	0.008	0.064	0.071	0.320
External	−0.133	0.090	−0.300	0.062	0.192	0.028	0.480
Ghrelin							
Awareness	0.000	0.040	−0.080	0.080	0.993	0.036	0.993
Distraction	−0.057	0.037	−0.132	0.018	0.134	0.089	0.383
Disinhibition	0.003	0.043	−0.080	0.090	0.948	0.036	1.000
Emotional	−0.064	0.036	−0.137	0.009	0.082	0.107	0.328
External	−0.129	0.041	−0.211	−0.046	0.003	0.229	0.060

Models adjusted for DII, parity, pregravid BMI, maternal age, and race.

DV, dependent variable; GWG, gestational weight gain; HOMA-IR, homeostasis model assessment of insulin resistance; MEQ, mindful eating questionnaire.

* Adjusted *p*-value using the false discovery rate method [33].

## Abbreviations:

BMI: body mass index
DII: dietary inflammatory index
GDM: gestational diabetes mellitus
GWG: gestational weight gain
HOMA-IR: homeostasis model assessment of insulin resistance
MEQ: mindful eating questionaire

## References

[1] Driscoll AK, Gregory ECW. Increases in Prepregnancy Obesity: United States, 2016–2019. NCHS Data Brief. 2020: 1–8. 33270551

[2] Chen C, Xu X, Yan Y. Estimated global overweight and obesity burden in pregnant women based on panel data model. PLoS ONE. 2018; 13: e0202183. 30092099 10.1371/journal.pone.0202183 PMC6084991

[3] Parrettini S, Caroli A, Torlone E. Nutrition and Metabolic Adaptations in Physiological and Complicated Pregnancy: Focus on Obesity and Gestational Diabetes. Frontiers in Endocrinology. 2020; 11: 611929. 33424775 10.3389/fendo.2020.611929 PMC7793966

[4] Barbour LA, Hernandez TL. Maternal Lipids and Fetal Over-growth: Making Fat from Fat. Clinical Therapeutics. 2018; 40: 1638–1647. 30236792 10.1016/j.clinthera.2018.08.007 PMC6195465

[5] Heerman WJ, Bian A, Shintani A, Barkin SL. Interaction between Maternal Prepregnancy Body Mass Index and Gestational Weight Gain Shapes Infant Growth. Academic Pediatrics. 2014; 14: 463–470. 25169157 10.1016/j.acap.2014.05.005 PMC4151184

[6] Tomedi LE, Simhan HN, Chang CH, McTigue KM, Bodnar LM. Gestational Weight Gain, Early Pregnancy Maternal Adiposity Distribution, and Maternal Hyperglycemia. Maternal and Child Health Journal. 2014; 18: 1265–1270. 24101436 10.1007/s10995-013-1361-3 PMC3980188

[7] Toro-Ramos T, Sichieri R, Hoffman DJ. Maternal fat mass at mid-pregnancy and birth weight in Brazilian women. Annals of Human Biology. 2016; 43: 212–218. 26392036 10.3109/03014460.2015.1032348

[8] Villar J, Cogswell M, Kestler E, Castillo P, Menendez R, Repke JT. Effect of fat and fat-free mass deposition during pregnancy on birth weight. American Journal of Obstetrics and Gynecology. 1992; 167: 1344–1352. 1442988 10.1016/s0002-9378(11)91714-1

[9] Murray S, Tulloch A, Gold MS, Avena NM. Hormonal and neural mechanisms of food reward, eating behaviour and obesity. Nature Reviews Endocrinology. 2014; 10: 540–552. 10.1038/nrendo.2014.91 24958311

[10] Muktabhant B, Lawrie TA, Lumbiganon P, Laopaiboon M. Diet or exercise, or both, for preventing excessive weight gain in pregnancy. Cochrane Database of Systematic Reviews. 2015; CD007145. 10.1002/14651858.CD007145.pub3 PMC9428894 26068707

[11] Rogozińska E, Chamillard M, Hitman GA, Khan KS, Thangaratinam S. Nutritional manipulation for the primary prevention of gestational diabetes mellitus: a meta-analysis of randomised studies. PLoS ONE. 2015; 10: e0115526. 25719363 10.1371/journal.pone.0115526 PMC4342242

[12] Fair F, Soltani H. A metareview of systematic reviews of lifestyle interventions for reducing gestational weight gain in women with overweight or obesity. Obesity Reviews. 2021; 22: e13199. 33459493 10.1111/obr.13199 PMC8047893

[13] Raab R, Michel S, Günther J, Hoffmann J, Stecher L, Hauner H. Associations between lifestyle interventions during pregnancy and childhood weight and growth: a systematic review and meta-analysis. International Journal of Behavioral Nutrition and Physical Activity. 2021; 18: 8. 10.1186/s12966-020-01075-7 PMC7792105 33413486

[14] Davidson KW, Barry MJ, Mangione CM, Cabana M, Caughey AB, Davis EM, Behavioral Counseling Interventions for Healthy Weight and Weight Gain in Pregnancy: US Preventive Services Task Force Recommendation Statement. Journal of the American Medical Association. 2021; 325: 2087. 34032823 10.1001/jama.2021.6949

[15] Shivappa N, Steck SE, Hurley TG, Hussey JR, Hébert JR. Designing and developing a literature-derived, population-based dietary inflammatory index. Public Health Nutrition. 2014; 17: 1689–1696. 23941862 10.1017/S1368980013002115 PMC3925198

[16] Shin D, Hur J, Cho E, Chung H, Shivappa N, Wirth MD, Pre-Pregnancy Body Mass Index is Associated with Dietary Inflammatory Index and C-Reactive Protein Concentrations during Pregnancy. Nutrients. 2017; 9: 351. 10.3390/nu9040351 PMC5409690 28368304

[17] Zhang Z, Wu Y, Zhong C, Zhou X, Liu C, Li Q, Association between dietary inflammatory index and gestational diabetes mellitus risk in a prospective birth cohort study. Nutrition. 2021; 87–88: 111193. 10.1016/j.nut.2021.111193 33774421

[18] Moore BF, Sauder KA, Starling AP, Hébert JR, Shivappa N, Ringham BM, Proinflammatory Diets during Pregnancy and Neonatal Adiposity in the Healthy Start Study. The Journal of Pediatrics. 2018; 195: 121–127.e122. 29217099 10.1016/j.jpeds.2017.10.030 PMC6363107

[19] Lindsay KL, Buss C, Wadhwa PD, Entringer S. Maternal Stress Potentiates the Effect of an Inflammatory Diet in Pregnancy on Maternal Concentrations of Tumor Necrosis Factor Alpha. Nutrients. 2018; 10: 1252. 10.3390/nu10091252 PMC6163870 30200631

[20] Lindsay KL, Buss C, Wadhwa PD, Entringer S. The Effect of a Maternal Mediterranean Diet in Pregnancy on Insulin Resistance is Moderated by Maternal Negative Affect. Nutrients. 2020; 12: 420. 10.3390/nu12020420 PMC7071160 32041106

[21] Lindsay KL, Buss C, Wadhwa PD, Entringer S. The Interplay between Maternal Nutrition and Stress during Pregnancy: Issues and Considerations. Annals of Nutrition & Metabolism. 2018; 70: 191–200. 10.1159/000457136 PMC6358211 28301838

[22] Warren JM, Smith N, Ashwell M. A structured literature review on the role of mindfulness, mindful eating and intuitive eating in changing eating behaviours: effectiveness and associated potential mechanisms. Nutrition Research Reviews. 2017; 30: 272–283. 28718396 10.1017/S0954422417000154

[23] Miller CK, Kristeller JL, Headings A, Nagaraja H, Miser WF. Comparative effectiveness of a mindful eating intervention to a diabetes self-management intervention among adults with type 2 diabetes: a pilot study. Journal of the Academy of Nutrition and Dietetics. 2012; 112: 1835–1842. 23102183 10.1016/j.jand.2012.07.036 PMC3485681

[24] Most J, Vallo PM, Gilmore LA, St Amant M, Hsia DS, Altazan AD, Energy Expenditure in Pregnant Women with Obesity does not Support Energy Intake Recommendations. Obesity. 2018; 26: 992–999. 29797559 10.1002/oby.22194 PMC5978753

[25] Most J, Amant MS, Hsia DS, Altazan AD, Thomas DM, Gilmore LA, Evidence-based recommendations for energy intake in pregnant women with obesity. Journal of Clinical Investigation. 2019; 129: 4682–4690. 10.1172/JCI130341 PMC6819141 31369400

[26] Most J, Marlatt KL, Altazan AD, Redman LM. Advances in assessing body composition during pregnancy. European Journal of Clinical Nutrition. 2018; 72: 645–656. 29748651 10.1038/s41430-018-0152-8 PMC6348859

[27] Matthews DR, Hosker JP, Rudenski AS, Naylor BA, Treacher DF, Turner RC. Homeostasis model assessment: insulin resistance and beta-cell function from fasting plasma glucose and insulin concentrations in man. Diabetologia. 1985; 28: 412–419. 3899825 10.1007/BF00280883

[28] Apolzan JW, Myers CA, Cowley AD, Brady H, Hsia DS, Stewart TM, Examination of the reliability and validity of the Mindful Eating Questionnaire in pregnant women. Appetite. 2016; 100: 142–151. 26879222 10.1016/j.appet.2016.02.025 PMC4814227

[29] Framson C, Kristal AR, Schenk JM, Littman AJ, Zeliadt S, Benitez D. Development and Validation of the Mindful Eating Questionnaire. Journal of the American Dietetic Association. 2009; 109: 1439–1444. 19631053 10.1016/j.jada.2009.05.006 PMC2734460

[30] Thomas DM, Navarro-Barrientos JE, Rivera DE, Heymsfield SB, Bredlau C, Redman LM, Dynamic energy-balance model predicting gestational weight gain. The American Journal of Clinical Nutrition. 2012; 95: 115–122. 22170365 10.3945/ajcn.111.024307 PMC3238455

[31] Martin CK, Nicklas T, Gunturk B, Correa JB, Allen HR, Champagne C. Measuring food intake with digital photography. Journal of Human Nutrition and Dietetics. 2014; 27: 72–81. 23848588 10.1111/jhn.12014 PMC4138603

[32] van Woudenbergh GJ, Theofylaktopoulou D, Kuijsten A, Ferreira I, van Greevenbroek MM, van der Kallen CJ, Adapted dietary inflammatory index and its association with a summary score for low-grade inflammation and markers of glucose metabolism: the Cohort study on Diabetes and Atherosclerosis Maastricht (CODAM) and the Hoorn study. The American Journal of Clinical Nutrition. 2013; 98: 1533–1542. 24153342 10.3945/ajcn.112.056333

[33] Benjamini Y, Yekutieli D. The control of the false discovery rate in multiple testing under dependency. Annals of Statistics. 2001; 1165–1188.

[34] National Center for Health Statistics, final natality data, 2017–2019. Available at: www.marchofdimes.org/peristats (Accessed: 12 July 2021).

[35] Benjamini Y, Hochberg Y. Controlling the False Discovery Rate: a Practical and Powerful Approach to Multiple Testing. Journal of the Royal Statistical Society: Series B. 1995; 57: 289–300.

[36] Daubenmier J, Moran PJ, Kristeller J, Acree M, Bacchetti P, Kemeny ME, Effects of a mindfulness-based weight loss intervention in adults with obesity: a randomized clinical trial. Obesity. 2016; 24: 794–804. 26955895 10.1002/oby.21396 PMC4898945

[37] Ruffault A, Czernichow S, Hagger MS, Ferrand M, Erichot N, Carette C, The effects of mindfulness training on weight-loss and health-related behaviours in adults with overweight and obesity: a systematic review and meta-analysis. Obesity Research & Clinical Practice. 2017; 11: 90–111. 27658995 10.1016/j.orcp.2016.09.002

[38] Skibicka KP, Hansson C, Alvarez-Crespo M, Friberg PA, Dickson SL. Ghrelin directly targets the ventral tegmental area to increase food motivation. Neuroscience. 2011; 180: 129–137. 21335062 10.1016/j.neuroscience.2011.02.016

[39] Disse E, Bussier A, Veyrat-Durebex C, Deblon N, Pfluger PT, Tschöp MH, Peripheral ghrelin enhances sweet taste food consumption and preference, regardless of its caloric content. Physiology & Behavior. 2010; 101: 277–281. 20515700 10.1016/j.physbeh.2010.05.017

[40] Khani S, Tayek JA. Cortisol increases gluconeogenesis in humans: its role in the metabolic syndrome. Clinical Science. 2001; 101: 739–747. 11724664 10.1042/cs1010739

[41] Goldstein RE, Wasserman DH, McGuinness OP, Lacy DB, Cherrington AD, Abumrad NN. Effects of chronic elevation in plasma cortisol on hepatic carbohydrate metabolism. American Journal of Physiology-Endocrinology and Metabolism. 1993; 264: E119–E127. 10.1152/ajpendo.1993.264.1.E119 8430780

[42] Ulrich-Lai YM. Self-medication with sucrose. Current Opinion in Behavioral Sciences. 2016; 9: 78–83. 26977424 10.1016/j.cobeha.2016.02.015 PMC4787559

[43] van Loenen MR, Geenen B, Arnoldussen IAC, Kiliaan AJ. Ghrelin as a prominent endocrine factor in stress-induced obesity. Nutritional Neuroscience. 2020; 1–12. 10.1080/1028415X.2020.1863740 33373270

[44] Azzam I, Gilad S, Limor R, Stern N, Greenman Y. Ghrelin stimulation by hypothalamic-pituitary-adrenal axis activation depends on increasing cortisol levels. Endocrine Connections. 2017; 6: 847–855. 29038331 10.1530/EC-17-0212 PMC5682420

[45] Dallman MF. Stress-induced obesity and the emotional nervous system. Trends in Endocrinology and Metabolism. 2010; 21: 159–165. 19926299 10.1016/j.tem.2009.10.004 PMC2831158

[46] McCullough LE, Miller EE, Calderwood LE, Shivappa N, Steck SE, Forman MR, Maternal inflammatory diet and adverse pregnancy outcomes: Circulating cytokines and genomic imprinting as potential regulators? Epigenetics. 2017; 12: 688–697. 28678596 10.1080/15592294.2017.1347241 PMC5687326

[47] Hutchinson AD, Charters M, Prichard I, Fletcher C, Wilson C. Understanding maternal dietary choices during pregnancy: the role of social norms and mindful eating. Appetite. 2017; 112: 227–234. 28179204 10.1016/j.appet.2017.02.004

[48] Grider HS, Douglas SM, Raynor HA. The Influence of Mindful Eating and/or Intuitive Eating Approaches on Dietary Intake: a Systematic Review. Journal of the Academy of Nutrition and Dietetics. 2021; 121: 709–727.e1. 33279464 10.1016/j.jand.2020.10.019

[49] Epel E, Laraia B, Coleman-Phox K, Leung C, Vieten C, Mellin L, Effects of a Mindfulness-Based Intervention on Distress, Weight Gain, and Glucose Control for Pregnant Low-Income Women: a Quasi-Experimental Trial Using the ORBIT Model. International Journal of Behavioral Medicine. 2019; 26: 461–473. 30993601 10.1007/s12529-019-09779-2 PMC6785577

[50] Fuentes Artiles R, Staub K, Aldakak L, Eppenberger P, Rühli F, Bender N. Mindful eating and common diet programs lower body weight similarly: Systematic review and meta analysis. Obesity Reviews. 2019; 20: 1619–1627. 31368631 10.1111/obr.12918

[51] Dunn C, Haubenreiser M, Johnson M, Nordby K, Aggarwal S, Myer S, Mindfulness Approaches and Weight Loss, Weight Maintenance, and Weight Regain. Current Obesity Reports. 2018; 7: 37–49. 29446036 10.1007/s13679-018-0299-6

[52] Robinson E, Aveyard P, Daley A, Jolly K, Lewis A, Lycett D, Eating attentively: a systematic review and meta-analysis of the effect of food intake memory and awareness on eating. the American Journal of Clinical Nutrition. 2013; 97: 728–742. 23446890 10.3945/ajcn.112.045245 PMC3607652

